# Zoonotic risk factors associated with seroprevalence of Ebola virus GP antibodies in the absence of diagnosed Ebola virus disease in the Democratic Republic of Congo

**DOI:** 10.1371/journal.pntd.0009566

**Published:** 2021-08-12

**Authors:** Anna Bratcher, Nicole A. Hoff, Reena H. Doshi, Adva Gadoth, Megan Halbrook, Patrick Mukadi, Kamy Musene, Benoit Ilunga-Kebela, D’Andre Spencer, Matthew S. Bramble, David McIlwan, J. Daniel Kelly, Daniel Mukadi, Placide Mbala Kingebeni, Steve Ahuka, Emile Okitolonda-Wemakoy, Jean-Jacques Muyembe-Tamfum, Anne W. Rimoin

**Affiliations:** 1 UCLA Fielding School of Public Health, Los Angeles, California, United States of America; 2 Institut National de Recherche Biomedicale, Kinshasa, DRC; 3 Kinshasa School of Public Health, Kinshasa, DRC; 4 Direction de lutte contre la maladie-Ministère de la santé Publique, Kinshasa, DRC; 5 Center for Genetic Medicine Research, Children’s National Medical Center, Washington DC, United States of America; 6 Department of Microbiology and Immunology, Stanford University, Stanford, California, United States of America; 7 School of Medicine, University of California, San Francisco, San Francisco, California, United States of America; University of Texas Medical Branch / Galveston National Laboratory, UNITED STATES

## Abstract

**Background:**

Ebola virus (EBOV) is a zoonotic filovirus spread through exposure to infected bodily fluids of a human or animal. Though EBOV is capable of causing severe disease, referred to as Ebola Virus Disease (EVD), individuals who have never been diagnosed with confirmed, probable or suspected EVD can have detectable EBOV antigen-specific antibodies in their blood. This study aims to identify risk factors associated with detectable antibody levels in the absence of an EVD diagnosis.

**Methodology:**

Data was collected from September 2015 to August 2017 from 1,366 consenting individuals across four study sites in the DRC (Boende, Kabondo-Dianda, Kikwit, and Yambuku). Seroreactivity was determined to EBOV GP IgG using Zaire Ebola Virus Glycoprotein (EBOV GP antigen) ELISA kits (Alpha Diagnostic International, Inc.) in Kinshasa, DRC; any result above 4.7 units/mL was considered seroreactive. Among the respondents, 113 (8.3%) were considered seroreactive. Several zoonotic exposures were associated with EBOV seroreactivity after controlling for age, sex, healthcare worker status, location, and history of contact with an EVD case, namely: ever having contact with bats, ever having contact with rodents, and ever eating non-human primate meat. Contact with monkeys or non-human primates was not associated with seroreactivity.

**Conclusions:**

This analysis suggests that some zoonotic exposures that have been linked to EVD outbreaks can also be associated with EBOV GP seroreactivity in the absence of diagnosed EVD. Future investigations should seek to clarify the relationships between zoonotic exposures, seroreactivity, asymptomatic infection, and EVD.

## Author summary

Ebola virus (EBOV) is spread through exposure to infected bodily fluids of a human or animal. While EBOV can lead to a severe disease, Ebola Virus Disease (EVD), it is possible for individuals to have anti-EBOV antibodies without ever getting sick with EVD. Seroreactivity (the detection of antigen-specific antibodies) suggests that that person has been exposed to EBOV or a similar virus in the past. Our study looked for seroreactive individuals who have never received an EVD diagnosis in four sites across the Democratic Republic of the Congo. Then we checked if animal exposures previously linked to EVD were more common among seroreactive individuals than non-seroreactive individuals. Among respondents from all four sites, 113 (8.3%) were seroreactive to EBOV. Additionally, contact with bats, rodents, and eating non-human primate meat were associated with seroreactivity, indicating these factors may be predictors of undocumented EBOV exposure events. These findings show that some EVD risk factors may be associated with EBOV seroreactivity without EVD diagnosis. Future research is needed to clarify the relationships between zoonotic exposures, seroreactivity, asymptomatic infection, and EVD.

## Introduction

Ebola virus (EBOV) is a zoonotic filovirus spread through exposure to infected bodily fluids or contact with an infected human or animal [1,2]. While EBOV ecology remains largely unknown, it is thought that outbreaks are initiated by zoonotic spillover events, where virus is passed from an infected animal to a human [3,4]. In several cases, outbreaks have been traced back to a single human-animal interaction, most commonly with a bat or non-human primate [4–8]. When the source is not traceable to a single zoonotic spillover event, outbreaks often originate in forested areas or with individuals who frequent forests [5]. Once introduced into the population, disease may then be propagated through human-to-human transmission by those who exhibit EVD symptoms [9].

For every spillover event that results in EVD, there are likely many EBOV exposure events that produce subclinical or asymptomatic EBOV infection and serologic response[10]; Studies conducted throughout sub-Saharan Africa have identified individuals with detectable levels of antibodies that recognize EBOV antigens, yet without a history of EVD diagnosis[11–17]. Several different hypotheses could explain this phenomenon: surveillance failures, undiagnosed EVD due to subclinical or asymptomatic infection [18], infection by another ebolavirus species, or infection by a less virulent, unidentified virus, which is antigenically cross-reactive with known ebolavirus species [5]. The latter hypothesis is supported by the recent discovery of Bombali virus, an ebolavirus that is not known to cause disease in humans [19,20]. Additionally, these antibodies could represent exposure to, but not infection by, EBOV, which could occur if an individual were exposed to inactivated virus or virus particles [21–23]. Thus, evidence of exposure to EBOV in those without a history of EVD may originate from several potential etiologies, but ultimately suggests contact between an individual and EBOV or an EBOV-like virus.

Though the existence of EBOV seroreactivity among individuals with no history of EVD is well-documented[11–17], minimal research characterizes risk factors associated with such seroprevalence. A small number of previous studies have focused on demographics or ecological predictors of seroprevalence, such as an individual’s occupation being located in forest vs. non-forested areas [16,24–27]. However, few studies consider the association between seroprevalence and contact with specific animals that may carry EBOV, such as bats, or activities that may lead to zoonotic exposure. These more precise predictors of spillover events may be identified using serosurveys in areas where EBOV is thought to exist in the environment, including regions at high risk for EVD outbreaks.

The Democratic Republic of the Congo (DRC) has experienced twelve EVD outbreaks in history, and therefore is an optimal region in which to study zoonotic exposures to EBOV[28]. This study of individuals throughout the DRC aims to identify associations between zoonotic exposures and seroreactivity to EBOV in the absence of diagnosed EVD. If EBOV seroreactivity in the absence of EVD is correlated with protective immunity, identifying its predictors could have significant contributions to understanding natural Ebola resistance in the DRC. Relationships detected in this analysis will be used to better understand zoonotic EBOV exposure and seroprevalence of EBOV GP antibodies in the DRC.

## Methods

### Ethics statement

Ethical approval was obtained at UCLA Fielding School of Public Health (IRB#15–000333 for those in Boende, Kabondo-Dianda, and Yambuku; IRB#16–001346 for those in Kikwit) and the Kinshasa School of Public Health (ESP/CE/038/2015 for those in Boende, Kabondo-Dianda, and Yambuku; IRB#ESP/CE/022/2017 for those in Kikwit). All participants provided written informed consent and had the right to refuse participation at any time.

### Study design

**Fig 1:**
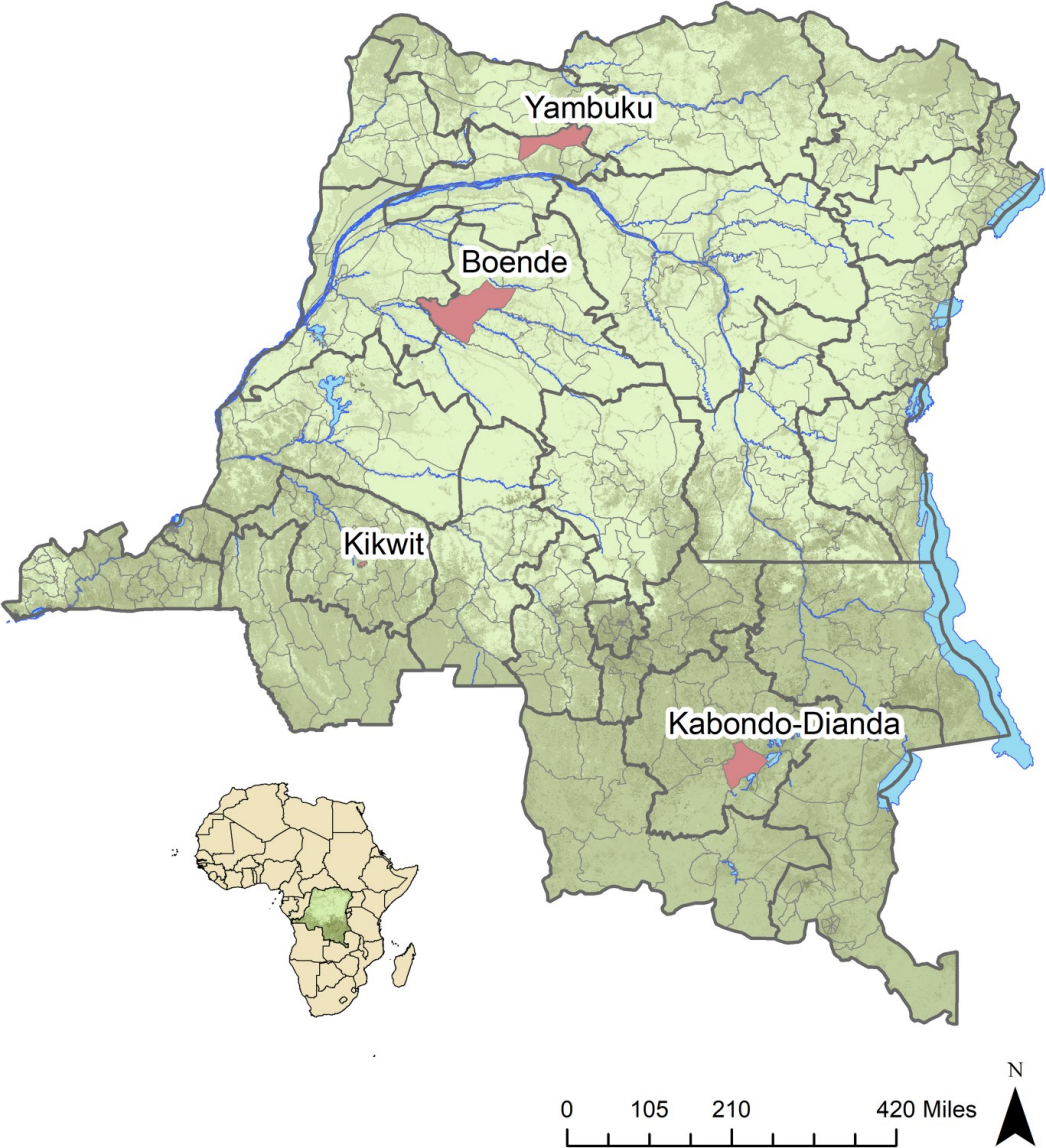
Map of the Democratic Republic of the Congo with health zones of study sites highlighted in red. Base layer from https://www.arcgis.com/home/item.html?id=10df2279f9684e4a9f6a7f08febac2a9.

As a part of a larger study examining EBOV serology across DRC, we conducted a cross-sectional substudy using data collected between September 2015 –August 2017. We enrolled 1,937 individuals across four sites around the country: 736 residents of Boende Health Zone, 41 residents of Kabondo-Dianda Health Zone, 424 residents of Yambuku Health Zone, and 736 residents of either North or South Kikwit Health Zones (Fig 1). Demographically, these regions are distinct in that Boende and Yambuku are in the north western part of the country, and considered very rural and heavily forested, Kikwit is semi-urban located in the central southwestern portion of the country and is considered forest-savannah mosaic and Kabando Dianda is semi-urban and located in the southeastern portion of the country also considered forest-savannah mosaic. Three of these areas have experienced previous EVD outbreaks (Boende, Kikwit, and Yambuku), while one has not had any known outbreak of EVD (Kabondo-Dianda). Participants were included regardless of self-reported presence at an EVD outbreak or self-reported exposure to an EVD case or if they lived in an area with a history of EVD.

Convenience samples were gathered at all four sites, targeting individuals who work in a healthcare setting, including traditional healers and pastors involved in healthcare activities, based on the WHO definition of healthcare worker [29]. However, eligibility was not restricted to individuals in those professions. Participants were eligible for the study if they were over 18 years of age and healthy, defined as no fever (<38°C) or other self-reported acute illness at the time of enrollment. At each site, a survey was administered and blood samples were collected from consenting participants at a single visit. Blood samples were obtained from all consenting participants by venipuncture using red-top Vacutainer tubes for serum collection (BD Biosciences).

Among the eligible 1,937 individuals, 1,407 (72.6%) had available serological results. Serological results were obtained according to assay availability at time of collection. Of these 1,407 participants, 41 had previously received an EVD diagnosis (2.9%) and were excluded from the analysis. None of the individuals in our study had received any Ebola vaccine at the time of data collection. Participants were included regardless of self-reported presence at an EVD outbreak site or self-reported exposure to an EVD case, or if they lived in an area with a history of EVD. Thus, 1,366 individuals were included in this analysis. Subsections of this data have been used in previous publications[30,31].

### Survey measurements

Our explanatory variables were zoonotic exposures, which included both animal exposures and activities that could lead to contact with an infected animal. Surveys were conducted by trained interviewers in the participant’s preferred local language (French, Lingala, Swahili, or Kikongo), and collected data on sociodemographic and epidemiologic characteristics, including potential zoonotic exposures to EBOV. Exposures were first assessed by determining if the participant had ever performed each activity or had contact with the specified animal. Those indicating a previous exposure were then asked if that exposure had occurred in the past month.

### Laboratory measurements

EBOV GP IgG seroreactivity was the primary outcome, determined using the manufacturer’s protocol for Zaire Ebola Virus Glycoprotein (EBOV GP antigen) ELISA kits (Alpha Diagnostic International, Inc.) at the National Institute for Biomedical research in Kinshasa, DRC. Details on methodology have been described elsewhere [31,32]. Samples were run in duplicate, and the average of the two results was used to determine seroreactivity. The manufacturer’s classification places any sample above 1.0 units/mL as reactive. However, this analysis considers results greater than 4.7 to be seroreactive in order to be conservative. This more stringent reactivity criteria has been shown to increase sensitivity to 96.7% and specificity to 97.7% for this assay [18]. Increased cutoffs for all EBOV antibody assays are commonly applied to Congolese cohorts, which are thought to exhibit high levels of assay cross-reactivity due to high rates of infectious disease within these populations [18,31,32]. No confirmatory assay or virus neutralization was used to rule out cross-reactivity as a source of positive results in reactive samples.

### Analysis

Descriptive statistics on sample characteristics and demographics were obtained for the full sample. Crude odds ratios (OR) for EBOV seroreactivity were then obtained for select sample characteristics. Adjusted ORs describing the associations between EBOV seroreactivity and ever having a zoonotic exposure were produced using logistic regression. To further explore the role of recent or frequent exposures, adjusted ORs were then obtained for each exposure in the past month among individuals who had ever had that exposure. This addition was made to distinguish possible risk from any exposure over a lifetime versus recent or frequent exposures, as it is possible that there may be a different biological response in both situations. For all ORs, a 95% confidence interval is provided; A 95% CI that did not cross the null value of 1.00 was considered to be evidence of an association. No corrections were made for multiple comparisons.

**Fig 2:**
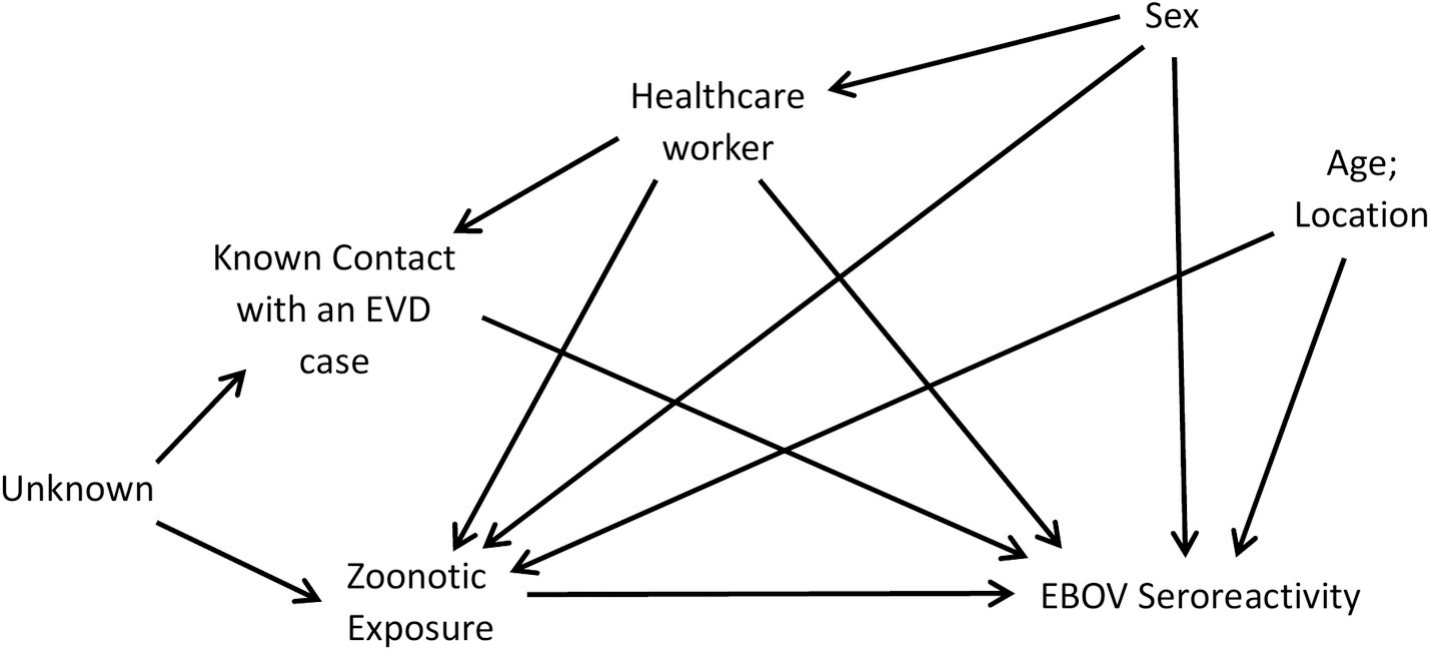
Directed Acyclic Graph (DAG) depicting hypothesized causal structure of zoonotic exposure and Ebola virus seroreactivity among individuals with no history of Ebola Virus Disease. Age and location are assumed to be simple confounders, influencing both presence of zoonotic exposures and Ebola virus (EBOV) seroreactivity. Sex is assumed to influence both zoonotic exposure and seroreactivity, and to be associated with healthcare worker status, as women were more likely to be healthcare workers in our sample. Being a healthcare worker is assumed to influence EBOV seroreactivity, due to potential for unknown exposures to Ebola Virus Disease (EVD) cases, and zoonotic exposure, due to relatively low contact with animals and forest when compared to non-healthcare workers. Working in healthcare is assumed to raise an individual’s risk of having known contact with an EVD case, which in turn is assumed to increase an individual’s risk of EBOV seroreactivity. Our DAG includes a residual confounding path through an unknown ancestor of zoonotic exposure and contact with an EVD case. In the analysis we controlled for contact with an EVD case as a proxy for this unknown confounder, which led to a more conservative analysis.

Adjusted ORs were obtained through multivariable logistic regressions that considered age as a continuous variable, sex, healthcare worker status, contact with an EVD case, and study site as confounders based on a priori assumptions depicted in our hypothesized directed acyclic graph (DAG) (Fig 2). Logistic regression was determined to be appropriate as there was independence of observations, linearity in the logit for continuous independent variables, an absence of multicollinearity, and an absence of influential outliers.[33] Additional analyses examining effect modification by study location were performed through the addition of interaction terms for each exposure by site to the specified models. A sensitivity analysis excluding all those with self-reported exposure to an EVD case was conducted. All statistical analyses were carried out using SAS software, version 9.4 (SAS Institute, Cary, NC).

## Results

Overall, 8.3% of our sample (113/1366) was seroreactive to EBOV GP. Most of our sample was male (62.6%) and between 26 to 59 years of age (78.7%). Nearly a third of our sample had finished primary school, and another third had completed secondary school as their highest level of education. Three quarters of participants (76.6%) were married or cohabitating with a partner, with only 15.4% reporting being single. Over two-thirds of the sample reported working in a healthcare setting, including traditional healers and pastors. A large majority (77.8%) reported that they had never had contact with a confirmed, suspected, or probable EVD case (Table 1).

**Table 1:** Sample Characteristics among 1,366 individuals from Boende, Kabondo-Dianda, Yambuku, and Kikwit in the Democratic Republic of the Congo, 2015–2017.

	Frequency (n)	Percent (%)
Sex		
*Male*	855	62.6
*Female*	511	37.4
Years of age^a^		
*18–25*	138	10.4
*26–35*	366	27.6
*36–45*	328	24.7
*46–59*	350	26.4
*60 or older*	145	10.9
Occupation^b^		
*Farmer*, *fisher*, *or hunter*	87	6.4
*Teacher*	31	2.3
*Healthcare worker*	959	70.6
*Merchant*	31	2.3
*Technician*	29	2.1
*Student*	22	1.6
*Driver*	14	1.0
*Other*	185	13.6
Type of healthcare worker^c^		
*Nurse*	402	42.1
*Physician*	25	2.6
*Room attendant*	117	12.2
*Midwife*	72	7.5
*Lab technician*	11	1.2
*Hygienic service*	92	9.6
*Traditional healer*	48	5.0
*Red cross worker*	24	2.5
*Administrator*, *supervisor*, *or epidemiologist*	68	7.1
*Other*	97	10.1
Education^c^		
*None*	41	3.0
*Started primary school*	106	7.8
*Finished primary school*	434	31.8
*Finished secondary school*	491	36.0
*Apprentice*	11	0.8
*College/University*	261	19.2
*Graduate school*	19	1.4
Marital status^d^		
*Single*	209	15.4
*Married*	1031	75.8
*Living together as married*	11	0.8
*Divorced or separated*	33	2.4
*Widowed*	76	5.6
Location		
*Boende*	687	50.3
*Kabondo-Dianda*	41	3.0
*Kikwit*	237	17.4
*Yambuku*	401	29.4
Has ever had contact with an Ebola Virus Disease case^e^		
*Yes*	270	19.8
*No*	1062	77.9
*Don’t know*	31	2.3
EBOV GP Reactive		
*Yes*	113	8.3
*No*	1253	91.7

a. 36 participants did not know their age; 3 missing responses

b. 8 missing responses

c. 3 missing responses

d. 6 missing responses

e. ‘Ebola Virus Disease case’ includes confirmed, probable, and suspected cases; 3 missing responses

In our sample, males were more commonly seroreactive. Meanwhile, a lower proportion of healthcare workers were seroreactive than non-healthcare workers. Proportionally more participants from Yambuku and Boende were seroreactive when compared to participants from Kabondo-Dianda, though not significantly. Both age and self-reported contact with a confirmed, suspected, or probably EVD case did not show meaningful associations with seroreactivity (Table 2).

**Table 2:** Seroreactivity to Ebola virus GP by select characteristics among 1,366 individuals from Boende, Kabondo-Dianda, Yambuku, and Kikwit in the Democratic Republic of the Congo, 2015–2017.

	Ebola virus GP reactive n = 113		Ebola virus GP non-reactive n = 1253			
	Frequency (n)	Percent (%)	Frequency (n)	Percent (%)	Crude odds ratio	95% confidence interval
Sex						
*Male*	90	10.5	765	89.5	*reference*	
*Female*	23	4.5	488	95.5	0.40	0.25, 0.64
Years of age^a^						
*18–25*	16	11.6	122	88.4	1.58	0.83, 3.03
*26–35*	28	7.6	338	92.4	*reference*	
*36–45*	26	7.9	302	92.1	1.04	0.60, 1.81
*46–59*	27	7.7	323	92.3	1.01	0.58, 1.75
*60 or older*	8	5.5	137	94.5	0.71	0.31, 1.59
Healthcare worker						
*Yes*	69	7.2	890	92.8	*reference*	
*No*	44	10.8	363	89.2	1.56	1.05, 2.33
Location						
*Boende*	48	7.0	639	93.0	1.47	0.34, 6.25
*Kabondo-Dianda*	2	4.9	39	95.1	*reference*	
*Kikwit*	8	3.4	229	96.6	0.68	0.14, 3.33
*Yambuku*	55	13.7	346	86.3	3.10	0.73, 13.20
Has ever had contact with an Ebola Virus Disease case^b^						
*Yes*	18	6.7	252	93.3	0.75	0.45, 1.27
*No*	92	8.7	970	91.3	*reference*	
*Don’t know*	3	9.7	28	90.3	1.13	0.34, 3.79

a. 36 participants did not know their age; 3 missing responses

b. “Ebola Virus Disease case’ includes confirmed, probable, and suspected cases; 3 missing responses

Among the animal exposures, those who had ever come into contact with a bat had 1.64 times (95% CI 1.06, 2.54) the odds of EBOV seroreactivity as those who had never had contact with a bat, holding confounders constant. Any contact with rodents was also associated with an increased odds of EBOV seroreactivity, as was eating non-human primate meat (Table 3).

**Table 3:** Adjusted odds ratios of seroreactivity to Ebola virus GP for various zoonotic exposures among individuals in the Democratic Republic of the Congo, 2015–2017.

	Exposed n (%)	Adjusted odds ratio^a^	95% confidence interval	
Has ever had contact with the following animals:				
*Monkeys*	893 (65.6)	1.34	0.82	2.19
*Other non-human primates*	293 (21.5)	1.50	0.90	2.50
*Bats*	468 (34.4)	1.64	1.06	2.54
*Rodents*	578 (42.5)	2.00	1.26	3.19
Has ever performed the following activities:				
*Slaughtered animals*	560 (41.1)	0.77	0.49	1.19
*Slept outside*	1009 (74.1)	1.51	0.85	2.68
*Hunted wild animals*	354 (26.8)	1.20	0.76	1.89
*Visited to wooded/forested areas*	1059 (77.8)	1.60	0.87	2.93
*Cut or collected firewood*	853 (62.8)	1.25	0.76	2.05
*Ate non-human primate meat*	468 (34.5)	1.64	1.01	2.65
*Frequented markets*	1130 (83.2)	1.54	0.79	3.01
*Made charcoal*	121 (8.9)	1.16	0.58	2.30
*Ate Bushmeat*	912 (67.0)	1.12	0.69	1.83
*Entered a cave or mine*	69 (5.1)	1.52	0.65	3.55

a. Adjusted for age, sex, healthcare worker status, known contact with an Ebola Virus Disease case, and study site

Among those who indicated they had ever had contact with a rodent, having contact with rodents in the past month was associated with a decrease in EBOV GP reactivity (Table 4).

**Table 4:** Adjusted odds ratios of seroreactivity to Ebola virus GP for recent zoonotic exposures among individuals who have ever experienced each respective exposure in the Democratic Republic of the Congo, 2015–2017.

	Adjusted odds ratio^a^	95% confidence interval	
Has had contact with these animals in the past month:			
*Monkeys*	0.63	0.40	1.04
*Other non-human primates*	0.61	0.25	1.48
*Bats*	0.91	0.48	1.75
*Rodents*	0.54	0.31	0.94
Has performed the following activities in the past month:			
*Slaughtered animals*	0.94	0.49	1.79
*Slept outside*	1.11	0.69	1.77
*Hunted wild animals*	1.29	0.98	2.53
*Visited to wooded/forested areas*	1.03	0.61	1.73
*Cut or collected firewood*	0.83	0.46	1.48
*Ate non-human primate meat*	1.45	0.78	2.69
*Went to a market*	0.88	0.47	1.66
*Made charcoal*^*b*^	-		
*Ate Bushmeat*	0.75	0.43	1.32
*Entered a cave or mine*	5.15	0.65	40.50

a. Adjusted for age, sex, healthcare worker status, known contact with an Ebola Virus Disease case, and study site

b. Estimate could not be obtained due to sparse data

There was no evidence of interaction in our models assessing effect modification of exposure effects by study site; all interaction terms were non-significant. Additionally, there were no significant or meaningful changes from the presented results when the analysis was restricted to those with no known EVD exposure.

## Discussion

In our study, we found associations between EBOV seroreactivity without diagnosed EVD and two zoonotic exposures previously tied to EVD outbreaks: contact with bats and eating non-human primate meat. These findings expand our understanding of zoonotic exposure, EBOV seroreactivity, and EVD risk within the DRC. Namely, it is possible that some exposures to bats or eating non-human primate meat in this region might result in EBOV seroreactivity without diagnosed EVD. If so, there may be contributing factors, such as environmental influences, chiropteran physiological conditions [34–36], or reduced virus viability in meat cooked for consumption[37], among these less consequential zoonotic interactions which result in low-consequence EBOV exposure. Future research should work to identify and evaluate potential variations in virus viability and viral load transfer associated with different types of human-bat interactions and non-human primate consumption to further describe EBOV zoonotic exposure and resulting risk of seroreactivity or EVD diagnosis.

Additionally, ever having rodent contact was associated with increased odds of seroreactivity in our sample, though rodent contact has never been implicated as a risk factor diagnosed EVD. The association between rodents and EBOV seroreactivity is plausible, given that the EBOV genome has been sequenced in samples from rodents in central Africa [8,38]. In contrast, those who had contact with rodents in the past month had significantly lower odds of seroreactivity than individuals who had contact with rodents that did not report contact in the last month. It is unclear why recent contact with rodents would have a protective effect. Future research should attempt to further understand the role of rodents in EBOV epidemiology.

In addition to zoonotic exposure findings, this analysis also describes EBOV seroreactivity across the DRC. A substantial portion of our sample, 8.3%, was seroreactive for antibodies to EBOV GP, with site specific seroreactivity in our sample ranging from 3.4% to 13.7%. Published EBOV seroprevalence estimates are greatly heterogeneous, varying widely depending on definition of reactivity, sample selection methods, and study area qualities such as ecology or rural versus urban settings [11]. Nevertheless, the estimates found in this study fall in line with previously reported seroprevalence estimates for comparable populations in sub-Saharan Africa [11–17,39]. Demographic predictors of EBOV seroreactivity in our sample were male gender and not being a healthcare worker. These findings contribute to our understanding of EBOV epidemiology, as there has been limited research into the demographic predictors of seroprevalence in the absence of EVD diagnosis for comparison. Other studies have identified higher seroprevalence in adults and women in similar cohorts [13,16,24].

While these findings are informative, this study has limitations. Our explanatory variables are self-reported and therefore may be subject to misclassification. Limitations of recall were addressed in part through the collection of reported exposure over the past month in addition to ever experiencing an exposure, with the assumption that recall over the previous month would be more accurate. Additionally, classification of serological results into a binary variable may not have accurately captured zoonotic exposure history. Our assay cut-off value is based on the manufacturer’s classification and previous research [31,32], and is reported to have 96.7% sensitivity and 97.7% specificity [18]. Given the cross-sectional study design, our findings are strictly associational. Further research, including collection and analysis of longitudinal data, will be vital to establishing temporality and uncovering any causal relationships behind the associations observed here.

This analysis provides new insights into the epidemiology of EBOV infection and disease in an endemic region of Central Africa. Our study is the first to evaluate associations between various zoonotic exposures and seroreactivity to EBOV GP in a large, geographically diverse sample of Congolese individuals who have never been diagnosed with EVD. This analysis demonstrates that some zoonotic exposures that have previously been implicated in EVD outbreaks can also be linked to EBOV GP seroreactivity in the absence of EVD diagnoses. Future investigations should seek to clarify the relationships between zoonotic exposures, seroreactivity, asymptomatic infection, and EVD.

## Supporting information

S1 Data Dataset used in this analysis. (XLSX)

S1 Codebook Variable names, corresponding survey questions, and values for the dataset used in this analysis. (XLSX)
