# Correction: Zoonotic risk factors associated with seroprevalence of Ebola virus GP antibodies in the absence of diagnosed Ebola virus disease in the Democratic Republic of Congo

**DOI:** 10.1371/journal.pntd.0010465

**Published:** 2022-05-18

**Authors:** Anna Bratcher, Nicole A. Hoff, Reena H. Doshi, Adva Gadoth, Megan Halbrook, Patrick Mukadi, Kami Musene, Benoit Ilunga-Kebela, D’Andre Spencer, Matthew S. Bramble, David McIlwain, J. Daniel Kelly, Daniel Mukadi, Placide Mbala Kingebeni, Steve Ahuka, Emile Okitolonda-Wemakoy, Jean -Jacques Muyembe-Tamfum, Anne W. Rimoin

The eleventh author’s name is spelled incorrectly. The correct name is: David McIlwain.
